# Radiation Exposure, Training, and Safety in Cardiology

**DOI:** 10.1016/j.jacadv.2024.100863

**Published:** 2024-02-29

**Authors:** Kamala P. Tamirisa, Mirvat Alasnag, Peter Calvert, Sabrina Islam, Anju Bhardwaj, Keerthana Pakanati, Shelley Zieroth, Mansour Razminia, Aarti S. Dalal, Mamas Mamas, Andrea M. Russo, Smadar Kort, ACC Women in Cardiology Advocacy Work Group

**Affiliations:** a Texas Cardiac Arrhythmia Institute, Austin, Texas, USA; b Cardiac Center, King Fahd Armed Forces Hospital, Jeddah, Saudi Arabia; c Liverpool Centre for Cardiovascular Science at University of Liverpool, Liverpool John Moores University and Liverpool Heart & Chest Hospital, Liverpool, UK; d Section of Cardiology, Lewis Katz School of Medicine, Temple University, Philadelphia, Pennsylvania, USA; e Advanced Cardiopulmonary Therapies and Transplantation, University of Texas/McGovern Medical School, Texas Medical Center, Houston, Texas, USA; f Center for Cardiovascular Health, Virginia Mason Franciscan Health, Seattle, Washington, USA; g Department of Cardiology, St. Boniface Hospital, Winnipeg, Manitoba, Canada; h Amita St. Joseph Hospital, Elgin, Illinois, USA; i Division of Cardiology, Department of Pediatrics, Vanderbilt Medical Center, Nashville, Tennessee, USA; j Keele Cardiovascular Research Group, Keele University, Stoke on Trent, UK; k Division of Cardiology, Cooper University Hospital, Camden, New Jersey, USA; l Department of Cardiology, Stony Brook Heart Institute, Stony Brook, New York, USA

**Keywords:** fluoroscopy, cardiology, pregnancy, radiation, safety, women in cardiology

## Abstract

Exposure to ionizing radiation is an inherent occupational health hazard in clinical cardiology. Health risks have been reported previously, including predilection to cancer. In addition, orthopedic injury due to prolonged wearing of heavy protective lead aprons, which are mandatory to reduce radiation risk, have been extensively documented. Cardiology as a specialty has grown with rising volumes of increasingly complex procedures. This includes electrophysiological, coronary, and structural intervention, advanced heart failure/transplant management, and diagnostic imaging. Both the operator as well imaging specialists are exposed to radiation, particularly in structural interventions where interventional cardiologists and structural imagers work closely. Increasingly, women interested in cardiology may deselect the field due to radiation concerns. This expert document highlights the risks of radiation exposure in cardiology, including practical tips within various subspecialty fields such as interventional/structural cardiology, electrophysiology, imaging, advanced heart failure, and pediatric cardiology.

## Highlights

• Cardiac procedures carry radiation risk.
• Practical tips across all subspecialties.
• Improve radiation safety with education/advocacy.

With advances in technology leading to percutaneous therapies for cardiovascular diseases, there has been an increase in radiation exposure to cardiologists working in the catheterization laboratory (cath lab).^1^

Prolonged exposure to ionizing radiation is associated with health hazards, including brain, head, and neck tumors,^2^ breast cancer,^3^ premature cataracts,^4^^,^^5^ thyroid disease, and early carotid atherosclerosis.^6^^,^^7^ Additionally, it may adversely affect the operator’s reproductive system.^3^^,^^8^

Radiation exposure is a major concern for women in their reproductive years, causing many to choose a different specialty. In an Italian survey in interventional cardiology, 56% of women were childless, and pregnancy was felt to be unsafe.^9^ This is one of the reasons for sex disparities in invasive cardiology.^10^ Women who do choose an invasive cardiology subspecialty may need to factor in risks of radiation exposure alongside family planning.

These concerns have garnered attention, and policies are being formulated to minimize radiation exposure and its sequelae.^11^, ^12^, ^13^

In this expert review, we will discuss the radiation risks inherent in interventional/structural cardiology, electrophysiology, imaging, advanced heart failure (AHF), and pediatric cardiology and provide practical recommendations for minimizing radiation exposure. Relevant terms used throughout this paper are defined in Table 1.

**Table 1:** Important Nomenclature Used Throughout This Manuscript

Term	Definition
Stochastic effects	Refers to certain adverse effects of radiation exposure, such as cancer. Stochastic effects occur by chance, with increasing probability at increased doses of exposure, however the severity of the outcome is independent of the dose.
Deterministic effects	Refers to other adverse effects of radiation exposure, such as cataracts and reproductive system effects. Unlike stochastic effects, deterministic effects only occur after a threshold dose is passed, and the severity of the effect increases with dose.
Fluoroscopy	X-ray imaging utilized during procedures for guiding equipment within the body. Fluoroscopy utilizes lower doses and lower frame rates to minimize radiation exposure to that which is required for safe manipulation of equipment.
Cine acquisition	Higher quality x-ray images, requiring higher doses and more rapid frame rates. Usually taken in short bursts to allow high quality images to be reviewed after acquisition, eg, coronary angiography.
Fluoroscopic time (min)	The duration during a procedure that fluoroscopy is active for. This does not include cine acquisition and tends to underestimate the total radiation dose when used in isolation.
Cumulative Air Kerma (Gy)	A measure of x-ray energy delivered to the air. Associated with deterministic skin effects.
Dose-area product (Gy/cm^2^)	Product of the radiation dose and beam area. This is an indicator of patient x-ray dose and is linked to the patient’s risk of stochastic effects.

## Coronary/structural cardiology

Radiation exposure during these procedures is substantial, especially in complex percutaneous coronary interventions (PCI) and structural interventions.^14^ Increased awareness and advances in protective measures have improved the response to radiation exposure. The PROTECTION VIII study, which included 3.7 million PCI procedures between 2008 and 2018 ^15^ showed that, over this period, radiation exposure was reduced by 36%.

Several factors are important when considering radiation exposure to the operator. The most important factor is patient body mass index (BMI), which independently increases operator radiation dose by approximately 5% per unit increase in BMI.^16^ With respect to equipment, older generation x-ray machines may not have the necessary quantum detection efficiency and calibration to reduce exposure with higher radiation doses. Operator behavior is a modifiable factor and includes appropriate image field definition and collimation, reduction in the frame rate, limited cine loop acquisition, compliance with protective measures and monitoring requirements, and maintaining maximal distance from the image intensifier.

All cath labs should have a ceiling-mounted, movable upper-body shield and lower-body shield mounted on the side of the patient table; these reduce scatter radiation by approximately 80% to 90%.^17^ There is variation in the effective use of these protection devices. While contemporary PCI practice has moved towards a radial-first approach, there is debate over whether the right or left radial approach is more optimal.^18^, ^19^, ^20^ Sciahbasi et al^21^ noted in their phantom simulation of diagnostic coronary angiography (DCoA) that operators had lower exposure at the wrist and thorax, higher radiation at hip level, and no difference at the head level when using left radial access. Another study detected similar radiation doses for operators at the body, shoulder, or thyroid level, with advantage at the wrist when using the left radial approach.^19^ Shah et al^20^ randomized operators to either right or left radial access and prospectively enrolled transfemoral procedures in a registry. Median measures of radiation exposure were not significantly different between left vs right radial in a population with high risk of transradial failure; however, all measurements of radiation were lowest in the transfemoral group. There remains a wide variation in practice, with the PROTECTION VIII study demonstrating a 5.3-fold variability of median dose area product between cath labs in Germany.^15^

Novel image enhancement technologies have been adopted to improve coronary stent visualization while minimizing x-ray duration. Tian et al^22^ evaluated the CLEARstent and CLEARstent Live (Siemens Healthineers, Germany) stent visualization modalities. They noted that enhanced imaging technology was associated with reduced contrast usage, radiation dose, and procedure time. The same results were observed in a subgroup of patients with chronic kidney disease.

While all personnel in the Cath lab should have their radiation exposure monitored with a dosimeter, standard dosimeters are limited by the lack of real-time updates on cumulative exposure. Operators may not be notified about their radiation dose for months until their dosimeter is reviewed. Dosimeters, which facilitate real-time radiation monitoring encourage staff to react accordingly and use strategies to limit exposure. Finally, technical advancements like robotic PCI may lower radiation exposure with similar overall fluoroscopy time, despite longer total procedural time.^23^ Adoption of such technologies has been limited due to technical and financial considerations.

### Practical tips

• Be aware of factors that increase x-ray exposure, such as patient’s BMI.
• Maximize distance from the image intensifier.
• Utilize novel stent visualization technology.
• Utilize standard radiation protection measures.

## Cardiac electrophysiology

### Catheter ablation

**Figure 1:**
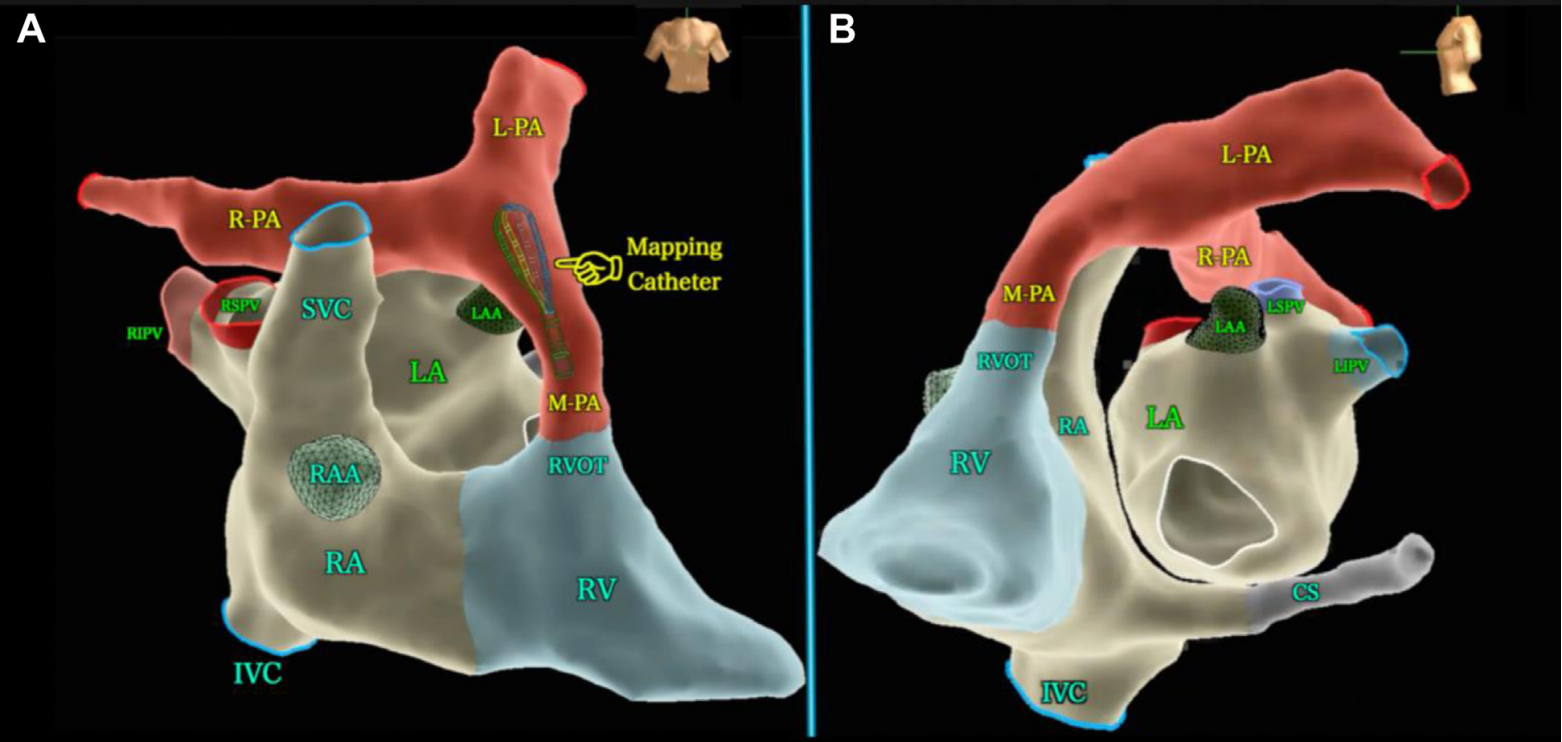
Illustration of an Electroanatomical Reconstruction of the Heart and Pulmonary Arteries in 2 Views Using Ensite X Cardiac Mapping System (A) AP view. Mapping catheter used to create the geometries is shown within the main pulmonary artery. (B) Left lateral view. IVC = inferior vena cava; LA = left atrium; LAA = left atrial appendage; LIPV = left inferior pulmonary vein; L-PA = left pulmonary artery; LSPV = left superior pulmonary vein; M-PA = main pulmonary artery; RA = right atrium; RAA = right atrial appendage; RIPV = right inferior pulmonary vein; R-PA = right pulmonary artery; RSPV = right superior pulmonary vein; RVOT = right ventricular outflow tract; SVC = superior vena cava.

**Figure 2:**
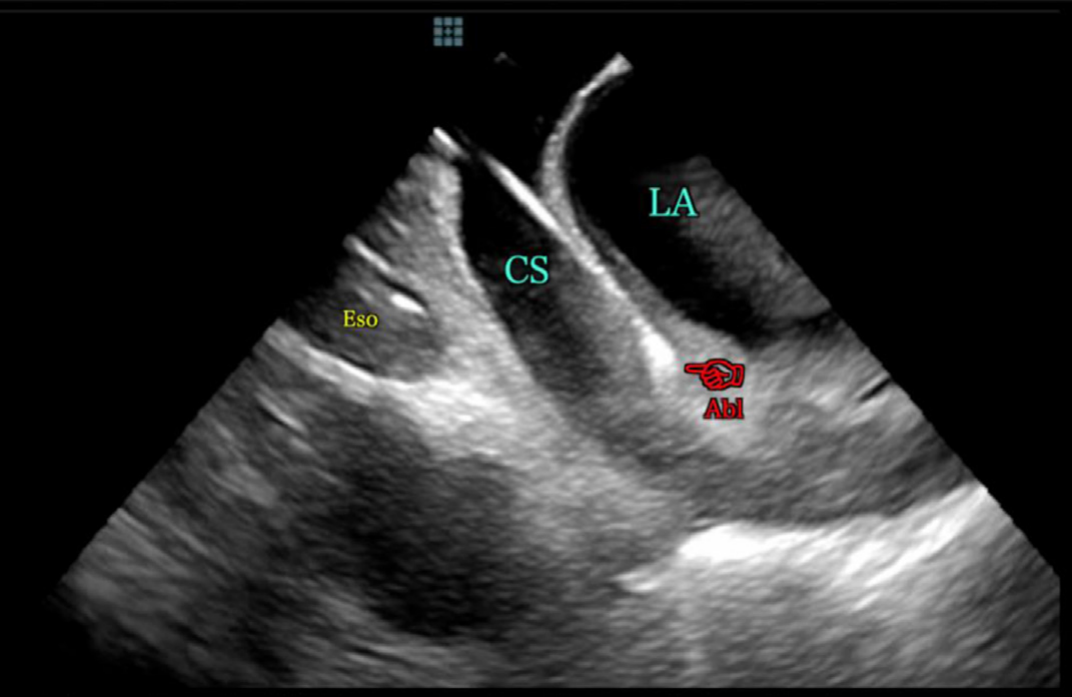
**Intracardiac Echocardiographic Image Showing Ablation Catheter Within the Coronary Sinus** Abl = ablation catheter; CS = coronary sinus; Eso = esophagus; LA = left atrium.

For many electrophysiology (EP) procedures, fluoroscopy guidance is used primarily for navigating tortuous vessels, performing transseptal punctures, and checking challenging catheter position. Recent advances in EP allow for “fluoroless” ablations without x-ray use, relying only on intracardiac electrograms, electro-anatomic mapping (EAM), and intracardiac echocardiography (ICE).^24^ Relying solely upon ICE and EAM allows one to maximize the potential of these imaging modalities and better define real-time cardiac anatomy as well as the tissue-catheter interface during catheter ablation (CA) while minimizing radiation exposure and the need for prolonged wear of lead aprons (Figures 1 and 2). It can allow pregnant patients to undergo CA safely,^25^ and it provides a safer environment for pregnant staff.

### Cardiac implantable electronic devices

Unlike CA procedures, shielding from x-ray exposure is limited for cardiac implantable electronic devices (CIED) implantation due to site of the procedure with proximity of the operator to the image intensifier, and absence of routine EAM platforms. Despite technological advancements, venous anatomy requiring venoplasty, CIED placement in patients with congenital cardiac disease, cardiac resynchronization therapy, and conduction system pacing may sometimes involve prolonged implantation procedures with increased radiation exposure. Case reports utilizing echocardiography, EAM, or stereotaxis have been described to minimize or achieve zero-fluoroscopy in special situations.^26^ Ultrasound-guided axillary venous access could potentially reduce fluoroscopy time.^27^

Factors impacting radiation exposure during CIED implantation include patient anatomy and body habitus, equipment characteristics and settings, and operator experience, behavior, and technique. Total fluoroscopy time is typically reduced with increased operator experience.^28^ Standard recommendations apply to CIEDs. Collimation to limit the size of the exposed field, pulse rate and acquisition frame rate, and position of the image intensifier and x-ray source, as well as total fluoroscopy time should be adjusted to minimize occupational exposure to “As Low As Reasonably Achievable (ALARA).” Operator shielding, including lead aprons, thyroid shields, leaded glasses, lead drapes under the table, ceiling-suspended shields when feasible, and leaded gloves may also be considered. Placement of radiation-absorbing drapes outside the beam path or use of a radiation protective cabin to reduce exposure to the operator’s head during long CIED cases may also be useful but are not routinely utilized.^29^^,^^30^ Although not always practical, limiting the maximum number of implants per year per physician may also be considered.

### Practical tips

• Maximize the use of EAM and ICE.
• Adopt fluoroless ablation.
• Prioritize fluoroless techniques during EP Training.
• Stereotaxis utilization, where available.
• Utilize ultrasound-guided access for CIED implant.

## Advanced heart failure/transplant cardiology

AHF procedures may involve right heart catheterizations (RHC) and endomyocardial biopsy (EMB). There is growing interest in the AHF community in pursuing diagnostic catheterization, placement of pulmonary artery pressure monitoring devices, trans-septal devices, intra-aortic balloon pumps, and other percutaneous hemodynamic support devices.

Until recently, physician radiation exposure during RHC and EMB was poorly understood. In a recent study,^31^ investigators observed that head-level radiation doses during RHC were lower than those observed with DCoA or PCI; however, radiation doses during EMB were like DCoA. They observed that a physician performing an RHC or EMB would receive more radiation than a physician performing DCoA when normalized to dose area product. This could be explained by 2 factors. Firstly, RHC and EMB operators utilize the internal jugular vein for access, decreasing distance from the radiation source and posing an increased risk of radiation scatter. It is known that intensity of radiation is inversely proportional to the square of the distance, and proximity to radiation source increases the expected radiation dose.^32^ Secondly, operator positioning required for manipulation of the catheter may preclude the use of ceiling-mounted shields, which have been demonstrated to decrease radiation exposure.^17^^,^^33^ RHC and EMB performed via femoral access require fluoroscopy for navigation of the inferior vena cava through to the pulmonary artery. There is evidence to suggest that using the ante-cubital vein for RHC access may reduce fluoroscopy time compared to more proximal veins.^34^

EMBs are technically difficult and have an increased risk of right ventricular perforation with no definitive way of confirming safe positioning of the bioptome; hence, most proceduralists confirm the position in multiple fluoroscopic projections, increasing cumulative exposure. For postcardiac transplant patients with multiple prior biopsies, there may be an extensive scar, and multiple attempts may be required to acquire tissue of suitable quality. Some operators have suggested the use of echocardiography to guide EMB, but data demonstrating its superiority over using fluoroscopic guidance remains inconclusive, and the trend has not caught on in many centers.^35^^,^^36^ One approach that may be underutilized is to perform EMB using EAM in the EP lab.^37^ This has multiple advantages, including reduced radiation exposure, improved diagnostic detail via voltage mapping, improved diagnostic yield by targeting substrate based on voltage, and potentially ablation of concurrent arrhythmia in the same procedure.

Insertion of a pulmonary artery monitoring device is similar to RHC, except that cineangiography of the pulmonary artery is needed to assess anatomy, and additional fluoroscopy is needed to confirm positioning/removal of the guiding catheter after implantation of the device.

### Practical tips

• Minimize fluoroscopy except where cardiac device dislodgement is a potential risk.
• Wedge positioning during RHC can be confirmed with obtaining a wedge saturation, which confirms position and ensures accuracy of the left-sided pressures.
• Limit the use of fluoroscopy with EMB by watching the bioptome using right anterior oblique projection and avoiding left anterior oblique projection.
• Consider the utility of EAM systems in the EP lab for EMB.

## Cardiac imaging

### Nuclear cardiology

Nuclear cardiology procedures utilize radioisotopes for visualization and assessment of myocardial function. Single-photon emission computed tomography myocardial perfusion imaging (SPECT MPI) utilizes 99mTc and 201Tl; radionuclide ventriculography utilizes 99mTc; and positron emission tomography utilizes 18F-FDG, 82Rb, and ammonia 13N.^38^ Radiation-induced injury can occur from gamma-ray emissions during SPECT MPI and radionuclide ventriculography or from positron emissions during positron emission tomography scans.

Health care personnel handling these radioisotopes are at risk of occupational exposure at multiple stages of the testing process, including the initial handling of these agents prior to patient administration and exposure to patients who become sources of radiation emission to their surrounding environment.^39^ The effective dose of radioisotopes administered determines the magnitude of risk from radiation and differs between the varying radioisotopes. For instance, the full-dose rest and stress protocol with 201Tl confers an effective dose of 21 mSv. In contrast, the full-dose stress-only protocol with 99mTc-sestamibi confers an effective dose of 10 mSv.^40^ Differing half-lives of radioisotopes determine how long the patient will emit radiation. Lastly, high-energy gamma particles released during nuclear testing are not as well shielded by standard protective equipment.^39^ Standard practices such as minimizing time and maximizing distance from the radiation source should be observed to decrease exposure. Additionally, strategies such as minimizing the effective dose administered to patients can reduce radiation emissions and overall exposure. Finally, separating patients from health care personnel and other patients immediately after receiving radioisotopes to allow for the radiotracers to undergo decay is essential for best safety practices. Table 2 summarizes the utility, half-life, and effective dose of several commonly used radioisotopes.

**Table 2:** Radioisotopes Commonly Used in Nuclear Cardiology Imaging Procedures

Radioisotope	Physical Half-Life	Procedure and Utility	Effective Dose
_99m_Tc-labeled agent^38^^,^^40^	6 h	SPECT MPI Myocardial perfusion, viability, function	^99m^Tc-sestamibi Stress only full dose: 10 mSv Rest and stress half dose: 6 mSv Rest and stress full dose: 13 mSv ^99m^Tc-tetrofosmin Rest and stress half dose: 6 mSv Rest and stress full dose 11 mSv
_201_ Tl^38^^,^^40^	73 h	SPECT MPI Myocardial perfusion, viability, function	Rest and stress half dose: 10.4 mSv Rest and stress full dose: 21 mSv
_82_Rb^38^^,^^40^	75 s	PET Myocardial perfusion, blood flow, function	3 mSv
_15_O-water^40^^,^^41^	2.1 min	PET Myocardial perfusion, blood flow, function	2 mSv
Ammonia _13_N^38^^,^^40^	10 min	PET Myocardial perfusion, blood flow, function	2 mSv
_18_F-Sodium fluoride^38^^,^^40^	110 min	PET Myocardial perfusion, blood flow, function	4 mSv
_18_F-FDG^38^^,^^40^	110 min	PET Myocardial metabolism, viability, inflammation	5 mSv

FDG = fluorodeoxyglucose; MPI = myocardial perfusion imaging; PET = positron emission tomography; Rb = rubidium; SPECT = single photon emission computed tomography; Tc = technetium; Tl = thallium.

### Interventional echocardiography

Advances in imaging technology have allowed for an increase in catheter-based procedures performed percutaneously under echocardiographic guidance. The field of echocardiography, traditionally considered an attractive field for women partially due to its radiation-free nature, has evolved, and interventional echocardiography has become a popular career path. With the requirement for echocardiographers to participate in fluoroscopic procedures, it is imperative for all practitioners to be aware of ionizing radiation exposure. These procedures are often performed in the cath lab or in hybrid operating rooms, which were not designed with the imaging specialist in mind. During these procedures, echocardiographers typically stand near the fluoroscopic image intensifier, where the greatest amount of radiation exposure occurs. Current designs often lack dedicated radiation shields for imagers. A recent study demonstrates significantly higher radiation exposure per case to echocardiographers than interventional cardiologists;^42^ this can be substantially reduced with proper cath lab modification and dedicated shields.^43^^,^^44^ Health care systems and professional organizations must help imagers by providing guidelines, education, and resources to minimize exposure.^45^

### Practical tips

• Additional shields should be utilized.
• Avoid ‘backless’ style lead aprons, as the interventional echocardiographer will frequently be facing away from the image intensifier.^43^
• Maximize distance from the image intensifier and leave the field when imaging guidance is not required.
• Be aware of the impact of x-ray projection–steep right anterior oblique imaging significantly increases radiation dose to the transesophageal echocardiographer.^43^

## Pediatric cardiology

To ensure safety, pediatric cardiac proceduralists must adhere to the same safety precautions as adult proceduralists. However, exposure to the patient is even more critical than in adult cardiology.

The practitioner should be aware of factors that may place the pediatric and congenital heart disease patient at higher risk of ionizing radiation. Exposure to ionizing radiation during periods of rapid cell growth and turnover places the young patient at higher risk of the detrimental effects of ionizing radiation. This is further exacerbated by the assumed longer life expectancy of the younger patient. Patients with congenital heart disease often have complex pathology, resulting in challenging structural anomalies and arrhythmias, leading to prolonged and repeated procedures. Thus, optimizing techniques to minimize radiation exposure is vital, as is monitoring the lifetime cumulative dose. Chosen hardware and provider practices should be optimized for pediatric patients. Patient positioning, shielding, age-appropriate dosing, and reducing radiation scatter are standard practices. The safe and effective use of low or no fluoroscopy during pediatric catheter procedures has been reported.^46^, ^47^, ^48^ Current technologies used in adult cardiology to minimize radiation exposure may not be feasible in this population due to the anatomic and size limitations. Therefore, using more traditional imaging such as transesophageal echocardiography should be considered to maintain safety and minimize radiation exposure.

### Practical tips

• Consider the cumulative exposure risk to the younger patient.
• Adopt low/no fluoroscopy and nonfluoroscopic imaging approaches where feasible.

## Orthopedic injury and strain

Use of heavy lead aprons, which provide radiation protection may result in orthopedic injury. This is particularly relevant when the operator stands for several hours, depending on the case duration and complexity. Several studies have shown that operators in cath lab suffer from spinal problems, which may cause them to take sick leave.^49^, ^50^, ^51^

The 2017 Society of Interventional Radiology document provided practical advice on reducing such injury;^52^ however, there are no specific cardiology society guidelines at the present time. These should be considered as a matter of importance. Using appropriately sized, low-weight lead aprons is important to minimizing orthopedic problems. Integrating lead-free environment into the current procedural laboratories using encapsulated patient table shielding systems (Egg Medical, Inc) or extended shielding systems (Rampart ic and Protego Systems) is another vital step toward preventing orthopedic injuries among the operators. Allowing time for regular breaks and ability to sit down during procedures is beneficial. Optimizing one’s own posture during procedures and regular leg movement may help to prevent joint stiffness and indeed reduce the risk of deep vein thrombosis, which is known to be elevated in those who spend prolonged periods standing. Regular exercise to improve and maintain muscular strength and flexibility may help to reduce the risk of injury.

### Practical tips

• Use low-weight lead aprons where available, and call upon organizations to acquire them if they are unavailable.
• Remove lead aprons and take breaks between cases.
• Maximize procedural use of nonfluoroscopic guidance.
• Avoid movements that cause discomfort, and be aware of one’s own posture.
• Advocate for and use lead-free systems.
• Exercises to maintain flexibility and strength may prevent long-term spinal problems.

## Pregnancy

The difficulty of family planning and pregnancy while being exposed to radiation may cause women to veer away from cardiology as a field. Recent estimates suggest that <10% of interventional cardiologists are female, and surveys have suggested that more than 25% of women see radiation exposure as a barrier to selecting interventional cardiology as a subspecialty.^53^

Generally, the risks of fetal exposure to ionizing radiation are highest at the beginning of pregnancy and decrease as the fetus matures.^54^ Doses of ≥100 mGy within the first 2 weeks of conception may result in abortion. Similar doses during the major periods of organogenesis (3-8 weeks) may result in growth retardation or malformation. There is a risk of impairment of intelligence quotient at doses from 120 to 200 mGy up to week 15, and at doses up to 500 mGy up to week 25. In the third trimester, the risks are lower and are considered like early childhood radiation exposure. These aspects are summarized in Table 3. Although these risks are frightening for the pregnant proceduralist to consider, several studies suggest that, with proper radiation safety measures, fetal x-ray exposure is essentially negligible.^53^ The standard radiation safety measures described earlier in this document all remain applicable during pregnancy; however, there are some additional considerations:

**Table 3:** Pregnancy and Radiation

Weeks of Pregnancy	Dangerous Dose of Radiation	Risks of Radiation	Proposed Radiation Safety (Can Be Used at Any Stage of Pregnancy)
2 wk	>100 mGy	Abortion	Reduce the fluoroscopy framerate. Avoid extreme left anterior oblique angulation. Increase distance from the image intensifier. Keep the detector close to the table. Avoid cine acquisition as much as possible. Use collimation. Avoid magnification.
3-8 wk	≥100 mGy	Malformation or growth retardation	
8-15 wk	120-200 mGy	Intelligence quotient reduction risk	
16-25 wk	500 mGy		
26 wk to delivery	Lowest risk period, similar to early childhood exposure	Malformation, growth retardation, fatal or nonfatal cancers including leukemia and solid tumors	

Table adapted from reference.^54^

Pregnant women may be at higher risk of orthopedic injury due to uterine gravidity combined with the weight of a lead apron. For this reason, lighter-weight lead aprons should be preferred, although a minimum of 0.5 mm lead equivalency is still advised.^53^ Dedicated pregnancy lead aprons should be made available by organizations to maximize safety and comfort. Alternatively, some may opt to wear an additional lead apron for abdominal protection,^53^^,^^54^ though this may increase the risk of orthopedic strain. Using suspended lead suits has been shown to decrease the radiation exposure and mitigate technical difficulties associated with the traditional ceiling-mounted radiation shields.^55^

Fetal dosimeter should be utilized during pregnancy. This allows real-time monitoring to ensure safety and provides reassurance.

Breastfeeding may be a concern for some when performing fluoroscopic procedures. Fortunately, there is no evidence that ionizing radiation creates any additional risk to the breastfed baby,^53^ although standard radiation protection measures should continue to be applied. If a pregnant cardiologist does not feel 'safe' in the cath lab despite the above-mentioned ways of mitigating risk to the fetus, an option to stay out of the cath lab during pregnancy must be provided. Program directors and employers should be supportive of this decision and should consider altering the schedule that will allow the pregnant trainee/cardiologist to perform noninvasive duties like research, outpatient patient management, or inpatient consultation services that do not require exposure to radiation.^56^

Other considerations may include availability of time for expressing and storing breast milk during prolonged procedures and the provision and duration of maternity leave; an in-depth discussion of these aspects is outside of the scope of our document.

Overall, most evidence suggests that, with appropriate precautions, pregnant cardiologists can safely continue procedures.^53^^,^^54^

### Practical tips

• Use pregnancy-specific lead aprons.
• Wear a fetal dosimeter.
• Ensure standard radiation safety measures are utilized.

Practical tips across all cardiac specialties are organized in a tabulated format for quick reference (Table 4).

**Table 4:** Summary of Radiation Safety Practical Tips

Radiation Exposure	Practical Tips
Coronary/structural cardiology	• Be aware of factors which increase x-ray exposure, such as patient BMI • Maximize distance from the image intensifier • Utilize novel stent visualization technology • Utilize standard radiation protection measures
Cardiac electrophysiology	• Maximize the use of EAM and ICE • Adopt zero-fluoro ablation • Prioritize fluoro-less techniques during EP training • Stereotaxis utilization where available • Utilize ultrasound-guided access for CIED implant
Advanced heart failure/transplant cardiology	• Minimize fluoroscopy except where cardiac device dislodgement is a potential risk • Wedge positioning during RHC can be confirmed with obtaining a wedge saturation, which confirms position and ensures accuracy of the left sided pressures • Limit use of fluoroscopy with EMB by watching the bioptome using right anterior oblique projection and avoid left anterior oblique projection • Consider the utility of EAM systems in the EP lab for EMB
Cardiac imaging	• Additional shields should be utilized • Avoid ‘backless’ style lead aprons, as the interventional echocardiographer will frequently be facing away from the image intensifier • Maximize distance from the image intensifier, and leave the field when imaging guidance is not required • Be aware of the impact of x-ray projection–steep right anterior oblique imaging significantly increases radiation dose to the transesophageal echocardiographer
Pediatric cardiology	• Consider the cumulative exposure risk to the younger patient • Adopt low/no fluoroscopy and nonfluoroscopic imaging approaches where feasible
Orthopedic injury and strain	• Use low-weight lead aprons where available, and call upon organizations to acquire these if unavailable • Remove lead aprons and take breaks between cases • Maximize procedural use of nonfluoroscopic guidance • Avoid movements which cause discomfort and be aware of one’s own posture • Advocate for and use lead-free systems • Exercises to maintain flexibility, and strength may prevent long-term spinal problems
Pregnancy	• Use pregnancy-specific lead aprons • Wear a fetal dosimeter • Ensure standard radiation safety measures are utilized • Option to avoid radiation if preferred without interruption of training (research, nonprocedural training, etc)

BMI = body mass index; CIED = cardiac implantable electronic devices; EAM = electroanatomic mapping; EMB = endomyocardial biopsy; ICE = intracardiac echocardiogram; RHC = right heart catheterization.

## Conclusions

While advances have been made, ionizing radiation exposure during cardiology procedures remains an area of concern for many, particularly women in cardiology. Maximizing safety requires both strict adherence to international guidelines and improvements in individual and organizational radiation safety culture.^57^ Following standard radiation safety measures is important (Central Illustration). The importance of using lead-free systems and correct posture, mobility, flexibility, and strengthening exercises to maintain spinal health should not be overlooked.

**Central Illustration:**
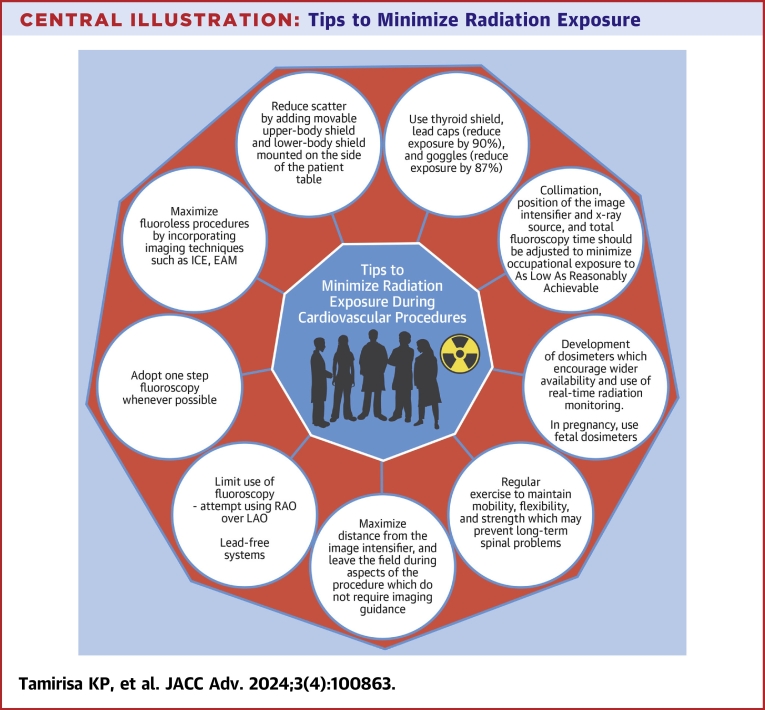
Tips to Minimize Radiation Exposure EAM = electroanatomic mapping; ICE = intracardiac echocardiography; LAO = left anterior oblique; RAO = right anterior oblique.

## Funding support and author disclosures

Dr Zieroth has received research grant support, served on advisory boards for, or speaker engagements with AstraZeneca, Bayer, BMS, Boehringer Ingelheim, Cytokinetics, Eli Lilly, GSK, Janssen, Merck, Novartis, Novo-Nordisk, Otsuka, Pfizer, Roche, Servier, and Vifor Pharma; and serves on a clinical trial committee or as a national lead for studies sponsored by AstraZeneca, Bayer, Boehringer Ingelheim, Merck, Novartis and Pfizer. Nonindustray support was received by Canadian Medical and Surgical KT Group, CCS, CHFS, Charite, EOCI, Liv, Medscape, Ology, PHRI, PACE-CME, Radcliffe, Translational Medicine Academy. Dr Dalal is a speaker for Medtronic. Dr Razminia is related to Abbott. All other authors have reported that they have no relationships relevant to the contents of this paper to disclose.
